# mTOR-Dependent and Independent Survival Signaling by PI3K in B Lymphocytes

**DOI:** 10.1371/journal.pone.0146955

**Published:** 2016-01-19

**Authors:** Mary Kaileh, Estefania Vazquez, Alexander W. MacFarlane, Kerry Campbell, Tomohiro Kurosaki, Ulrich Siebenlist, Ranjan Sen

**Affiliations:** 1 Gene Regulation Section, Laboratory of Molecular Biology and Immunology, National Institute on Aging, National Institutes of Health, Baltimore, Maryland, United States of America; 2 Laboratory of Molecular Immunology, National Institute of Allergy and Infectious Diseases, National Institutes of Health, Bethesda, Maryland, United States of America; 3 Fox Chase Cancer Center, Division of Basic Science, Institute for Cancer Research, Philadelphia, Pennsylvania, United States of America; 4 Laboratory of Lymphocyte Differentiation, WPI Immunology Frontier Research Center, Osaka University, Suita, Osaka, Japan; COCHIN INSTITUTE, Institut National de la Santé et de la Recherche Médicale, FRANCE

## Abstract

Peripheral B lymphocyte survival requires the B cell receptor (BCR) and B cell activating factor (BAFF) binding to its receptor (BAFF-R). Deletion of the BCR, or its signal transducing chaperone Igβ, leads to rapid loss of mature B cells, indicating that signals initiated at the BCR are crucial for B cell survival. BAFF or BAFF-R deficiency also significantly reduces the numbers of mature B cells despite normal BCR expression. Together, these observations indicate that continued BCR and BAFF-R signaling are essential for the survival of mature resting B cells in the periphery. Here we demonstrate that tonic BCR signals up-regulate p100 (Nfkb2) as well as Mcl-1 protein expression at a post-transcriptional level via a PI3K-dependent pathway. p100 expression is mTOR-independent, whereas Mcl-1 expression is mTOR-dependent. BAFF treatment further elevated Mcl-1 levels by an mTOR-independent pathway, while consuming p100. Accordingly, Mcl-1 induction by BAFF is abrogated in *Nfkb2*^*-/-*^ B cells. We propose that the cumulative effects of the BCR and BAFF-R signaling pathways increase Mcl-1 levels beyond the threshold required for B cell survival.

## Introduction

B lymphocyte development in the bone marrow produces immature cells that express unique immunoglobulin (Ig) molecules on the cell surface. However, these cells are not competent to mount immune responses, in part because activation via B cell receptor (BCR) leads to cell death and/or receptor editing, rather than generation of effector function. Immature B cells gain the ability to effectively respond to antigen during additional differentiation steps in the spleen [1,2]. Follicular B cells that result from peripheral differentiation are essential to mount T-dependent humoral responses, while marginal zone B cells are first-responders to blood borne pathogens. Antigen encounter by these mature B cell results in productive immune responses. In the absence of antigen recognition B lymphocytes ultimately die with a half-life of 5–6 weeks [3].

Two cell surface receptors are essential to maintain the mature B cell pool; one is the BCR and the other is the receptor for the cytokine, BAFF/ BLyS (BAFF-R). Deletion of the BCR from the surface of mature B cells leads to rapid loss of these cells from peripheral compartments without any effect on precursor B cell populations in the bone marrow, indicating that continued expression of the BCR is required for mature B cell survival [4,5]. Because conditional deletion of BCR-associated signal transducing proteins, such as CD79α and β, also deplete mature B cells from the periphery [5], one view is that B cell viability is maintained by antigen-independent survival signaling initiated at the BCR. This form of signaling has been referred to as ‘tonic’ signaling [6–9]. Recent studies with Nur77-GFP reporter mice show that the majority of follicular mature B cells exhibit some GFP expression, indicative of BCR signaling *in vivo* [10]. Further, the spleen tyrosine kinase, Syk, has also been shown to be essential for peripheral B cell viability, consistent with a role in tonic BCR signaling [11]. Biochemical basis of tonic BCR signaling remains enigmatic at best, especially because it has been proposed that the effects of deleting BCR-associated signaling components CD79α/β or Syk may be due to ineffective BAFF/BAFF-R signaling [11].

What is clear, however, is that most of the requirement for BCR-dependent mature B cell survival is recapitulated by constitutive activity of phosphatidyl inositol 3-kinase (PI3K). Using elegant genetic analyses, Srinivasan *et al*. showed that loss of B cells caused by deleting the BCR could be rescued by concurrent expression of active PI3K [12]. Importantly, the resulting double- mutant B cells required BAFF/BAFF-R signaling for survival at least *ex-vivo*, indicating that PI3K activity circumvented only BCR-initiated signals, but not BAFF-R initiated signals. How PI3K activity contributes to B cell survival via a BCR-dependent pathway remains to be elucidated.

Observations with BAFF- or BAFF-R-deficient mice mirror those of BCR deficiency. Mature B cells are absent in mice that lack either BAFF-R or BAFF, and depletion of circulating BAFF in normal mice depletes mature peripheral B cells without affecting bone marrow populations [13–17]. BAFF/BAFF-R interactions activate several downstream signaling pathways: classical NF-κB (consisting of p50/RelA heterodimer) is induced transiently [18], alternative NF-κB (consisting of p52/RelB complexes) is induced persistently, and PI3K is activated after extended treatment of splenic B cells with BAFF [19–22]. Two observations contributed to a view that the alternate NF-κB pathway provided much of the basis for BAFF-dependent B cell survival. First, deficiencies in either NIK or IKK1 (the predominant kinases regulating the alternative NF-κB pathway), and BAFF or BAFF-R blocked B cell development at the immature stage [23–26], and second, *Nfkb2*^*-/-*^ mature B cells were not responsive to BAFF-dependent survival signal *in vitro* (S1A and S1B Fig) [20]. However, recent studies demonstrate that conditional deletion of IKK1 in mature B cells has very little effect on B cell homeostasis, indicating a more complex relationship between BAFF/BAFF-R, IKK1 and the alternative NF-κB pathway [27]. Though PI3K activity is widely associated with cell survival, and B cells that lack p110α/δ subunits of PI3K are blocked at the immature stage [28,29], the significance of BAFF-induced PI3K remained unclear until Jellusova *et al*. demonstrated that deletion of the gene encoding phosphatase and tensin homolog (PTEN), a negative regulator of PI3K, rescues mature B cell deficiency in BAFF^-/-^ mice. Taken together with the results of Srinivisan *et al*., these observations indicate that both critical cell surface receptors involved in mature B cell survival can be substituted by sustained activation of PI3K.

An intriguing question that arises from these studies is why two receptors have evolved to activate the same (PI3K) pathway to maintain B cell viability. One possibility is that each receptor functions independently to provide survival signals and their cumulative effect exceeds the threshold needed for survival. Alternatively, the receptors may cross-talk; that is, a consequence of signaling through one receptor impacts the function of the second receptor and vice-versa. Both these models require BCR-initiated signals for cell survival. We have previously advocated a cross-talk model based on the observation that tonic BCR signals induce p100 (Nfkb2), which is essential to activate the alternative NF-κB pathway by BAFF [7]. However, Schweinghoffer *et al*., recently proposed that the BCR serves only as a scaffold for Syk activation by BAFF-R, rather than as a signaling model *per se* [11]. In this model, BAFF/BAFF-R interactions are sufficient for B cell viability.

In addition to p100, BAFF-dependent B cell survival requires the anti-apoptotic Bcl-2 homolog, Mcl-1. Woodland *et al*. demonstrated that BAFF treatment induced Mcl-1 expression in splenic B cells and, conversely, Mcl-1-deficient B cells did not survive in response to BAFF [30]. Two signaling pathways, requiring either Pim2 or PI3K activated mTOR, contributed redundantly to Mcl-1 induction by BAFF. Accordingly, neither Pim2-deficiency alone nor rapamycin treatment alone inhibited BAFF-induced Mcl-1 expression, or affected B cell viability in response to BAFF. However, Mcl-1 induction and B cell survival, was attenuated in Pim2-deficient B cells that were treated with BAFF in the presence of rapamycin. This two- signal survival pathway cannot easily be fitted into a model where BAFF-induced activation of PI3K is sufficient for B cell survival.

To better understand the mechanisms that regulate mature B cell homeostasis, we further tested the notion of tonic BCR signaling *ex vivo*. Our working hypothesis was that signaling consequences that were observed in the absence of ectopic BAFF administration could not have been initiated at the BAFF-R. Furthermore, if these consequences partially mimicked effects of acute BCR activation, then the simplest interpretation would be that they were the result of “tonic” BCR signaling. We provide evidence that both key molecules that transmit BAFF-R survival signals, p100 and Mcl-1, are induced by tonic (and acute) BCR signaling at a post-transcriptional level via involvement of PI3K. Despite sharing initiation (at the BCR) and transduction (via PI3K) pathways, the mechanism by which each protein was induced diverged at the final step; while p100 induction was mTOR-independent, Mcl-1 induction was mTOR-dependent.

Having provided new evidence for tonic BCR signaling, we reasoned that the effects of BAFF treatment *ex vivo* must be interpreted in the context of concurrent tonic BCR signaling. We found that in BAFF-treated B cells Mcl-1 expression was induced via mTOR-dependent and mTOR-independent pathways. The mTOR-dependent pathway likely initiates at the BCR. The mTOR-independent pathway likely proceeds via p100 which we found to be essential for Mcl-1 induction by BAFF. Taken together, we propose that mTOR-dependent and independent pathways activate key survival molecules downstream of the BCR that further synergize with BAFF-R-initiated signals to maintain B cell viability.

## Results

Both BCR and BAFF-R are necessary to maintain B cell viability *in vivo*. To understand the signaling mechanisms involved, it was essential to dissociate the consequences of signals initiated at each receptor. However, as the loss of either receptor leads to drastic reduction in the numbers of mature B cells, this is difficult to do *in vivo*. For experiments carried out *ex vivo*, tonic BCR signaling remains a confounding factor in the dissection of signaling mechanisms exclusive to BAFF-BAFF-R. Specifically, if the BCR emits persistent signals, then the effects observed after BAFF treatment must be a consequence of both BCR and BAFF-R-initiated signals. If, however, there is no tonic BCR signaling, the consequences of BAFF treatment can be assumed to be only due to signals initiated at the BAFF-R. To distinguish between these possibilities it was essential to seek experimental evidence in favor of, or against, tonic BCR signaling.

### mTOR-dependent induction of Mcl-1 expression by the BCR

**Fig 1:**
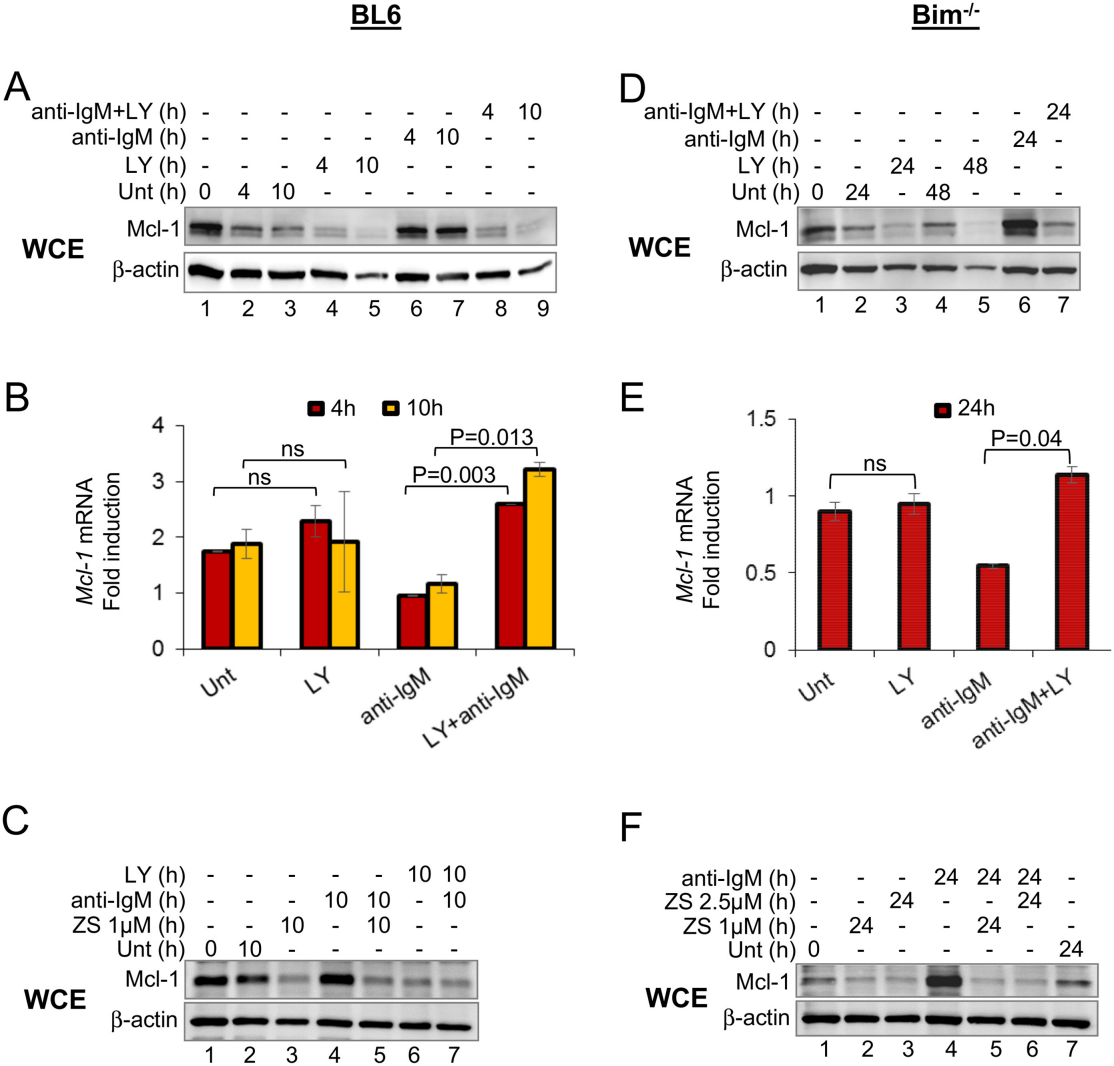
Role of PI3K pathway in Mcl-1 up-regulation by the BCR. CD43^-^ splenic B cells from BL6 (A-C) or Bim^-/-^ (D-F) mice were left untreated or treated with anti-IgM F(ab’)_2_ (15μg/ml) for various times as indicated, in the presence or absence of the PI3K inhibitor LY294002 (20μM) or ZSTK474 (ZS, 1μM and 2.5μM). (A, C, D, F) Whole cell extracts were fractionated by SDS-PAGE and Mcl-1 expression has analyzed by immunoblotting. β-actin was used to normalize between samples. (B, E) Mcl-1 mRNA levels were determined by quantitative RT-PCR; β-actin served as a normalizing control between samples. mRNA fold-change (Y axis) was calculated relative to the mRNA levels in untreated cells at 0h. The average of 3 independent experiments is shown. Error bars represent the standard error of the mean between experiments. P values were calculated using paired TTEST in Microsoft Office Excel (2013) with two tailed distribution. Ns = not significant. Cell viability profile for all conditions are shown in S3 Fig.

We previously proposed that up-regulation of p100 in primary B cells cultured *ex vivo* was a consequence of tonic BCR signaling [7]. To obtain further evidence in support or against the contribution of tonic BCR signaling to B cell viability, we examined pathways involved in modulating two key survival proteins, p100 and Mcl-1, in *ex vivo* cultured naïve splenic B cells. We first tested the role of PI3K as constitutive activity of this kinase has been shown to compensate the loss of either BCR or the BAFF-R [12,27]. We found that Mcl-1 expression in B cells was maintained by a PI3K-dependent pathway (Fig 1A, lanes 1–5), based on reduced levels of Mcl-1 in cells that had been treated with the PI3K inhibitor LY294002 (LY) for 4 and 10h compared to the untreated control. Maintenance of Mcl-1 expression at these time points (Fig 1A, lane 2, 3) represented newly synthesized protein, as cycloheximide treatment abolished Mcl-1 expression (S2A Fig). Since Mcl-1 mRNA levels did not correlate with Mcl-1 protein levels (Fig 1B), we concluded that PI3K regulated Mcl-1 expression at the translational level. To confirm that this effect was mediated by PI3K we used ZSTK474 (ZS) a second, more specific, PI3K inhibitor in this assay. Mcl-1 expression in cells incubated for 10h *ex vivo* was reduced in the presence of ZS (Fig 1C, lanes 1–3). Similar results were obtained with cells that had been cultured overnight to remove any pre-bound BAFF that may have co-purified with the B cells (S2B and S2C Fig).

Because these cells had not been treated with BAFF, nor was there any evidence that they purified with pre-bound BAFF, our interpretation is that PI3K-dependent Mcl-1 expression was a consequence of BCR signaling. To test this idea, we activated B cells by cross-linking the BCR using anti-IgM; Mcl-1 levels were substantially induced by anti-IgM-treatment (Fig 1A, lanes 2, 3, 6, 7) in a PI3K-dependent manner (Fig 1A, lanes 6–9; Fig 1C, lanes 4–7). Anti-IgM treatment did not increase Mcl-1 mRNA (Fig 1B) compared to untreated control, consistent with the idea that induced PI3K activity regulated Mcl-1 translation.

Due to the extreme sensitivity of splenic B cells to PI3K inhibition (S3A and S3B Fig), we carried out similar experiments with B cells isolated from Bim-deficient mice [31,32], which can be cultured for up to 48h without significant loss of viability (S3C and S3D Fig). We found that Mcl-1 expression in unactivated cells after 24h or 48h of culture required PI3K activity (Fig 1D, lanes 1–5). Similarly, anti-Ig-induced Mcl-1 expression was also abolished in LY-treated cells (Fig 1D, lanes 6, 7). Again, these changes were not reflected in Mcl-1 mRNA levels (Fig 1E). Anti-IgM-induced Mcl-1 expression was also abolished by inclusion of the PI3K inhibitor ZS (Fig 1F). We conclude that maintenance of Mcl-1 expression or its induction via BCR crosslinking occurs by a post-transcriptional mechanism that requires PI3K activity.

**Fig 2:**
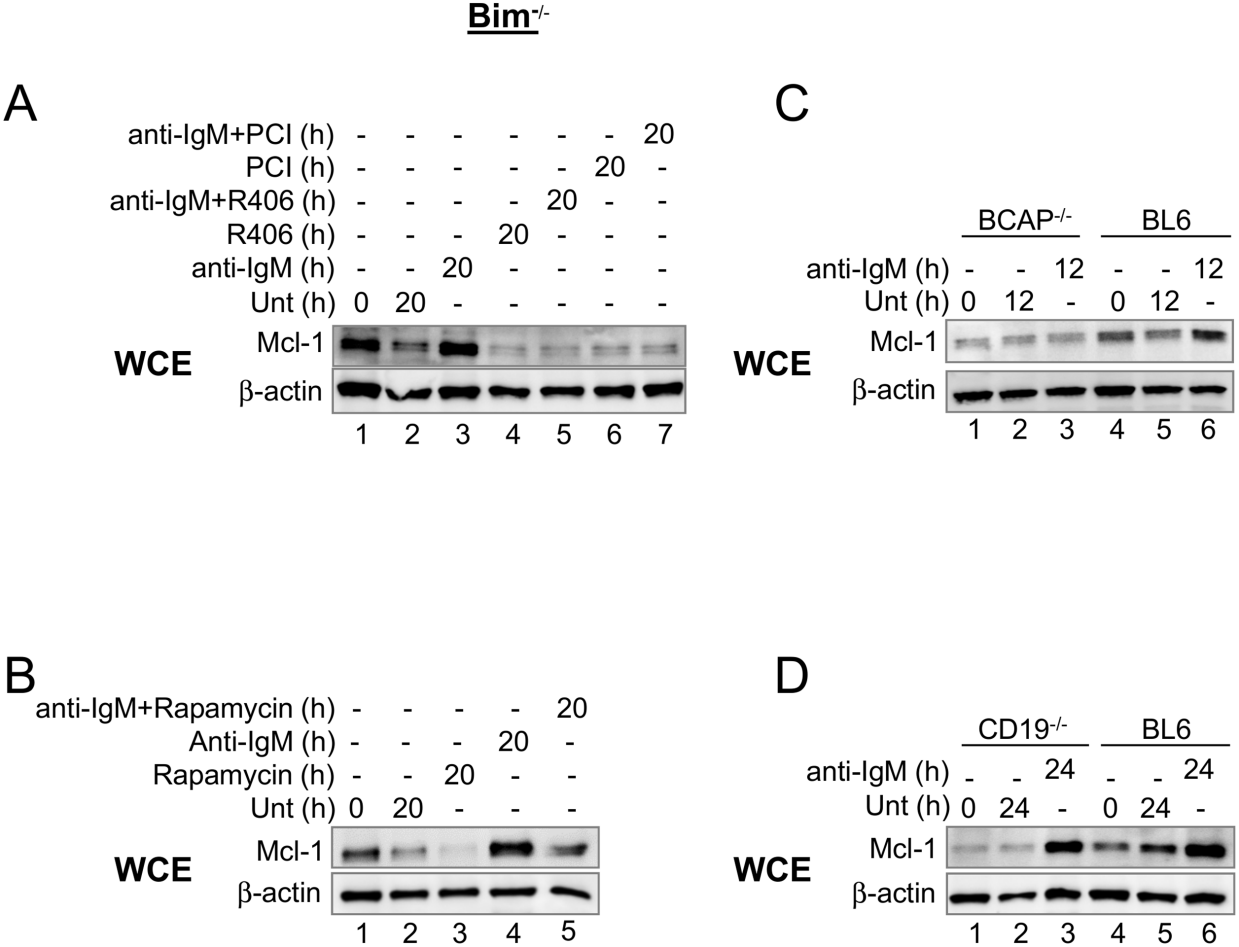
Signaling pathway to BCR-induced Mcl-1 expression. (A, B) CD43^-^ splenic B cells from Bim^-/-^ mice were treated with anti-IgM F(ab’)_2_ (15μg/ml) for various times in the presence or absence of Syk kinase inhibitor R406 (4μM), Bruton’s tyrosine kinase inhibitor Ibrutinib (PCI-32765) (20nM), or TORC1 inhibitor rapamycin (50nM). Whole cell extracts were fractionated by SDS-PAGE and Mcl-1 protein was analyzed by immunoblotting. β-actin was used as a loading control to normalize between samples. (C, D) CD43^-^ splenic B cells from BL6, BCAP^-/-^ (C) or CD19^-/-^ (D) mice were cultured for the indicated times with or without anti-IgM F(ab’)_2_ (15μg/ml). Whole cell extracts were prepared and assayed for Mcl-1 levels by Western blot analysis. β-actin was used as a loading control to normalize between samples. Representative gels from 3 independent experiments are shown for A and B and 2 independent experiments for C and D. Cell viability profile of BCAP^-/-^ and CD19^-/-^ B cells are shown in S4 Fig.

To study signaling pathways required for Mcl-1 expression in *ex vivo* cultured B cells, or upon BCR crosslinking, we used specific pharmacologic inhibitors. Inhibition of Syk kinase with R406 [33] (Fig 2A, lanes 1–5), Bruton’s tyrosine kinase (Btk) using PCI-32765 [34] (Fig 2A, lanes 1–3, 6, 7), or mTOR with rapamycin (Fig 2B) attenuated Mcl-1 expression in cultured or anti-Ig-treated B cells. Close coincidence between signaling components required to maintain Mcl-1 expression, or induce expression by anti-Ig, supported the idea that maintenance was the consequence of low-grade activation of the BCR. Our interpretation is that this is a signature of ‘tonic’ BCR signaling. BCR induced PI3K activation has been previously shown to “redundantly” require either the B cell adapter protein (BCAP) or the co-receptor, CD19 [35–40]. We found that Mcl-1 was not induced in BCAP-deficient B cells in response to BCR crosslinking (Fig 2C and S4A Fig), whereas its expression was unaffected in CD19-deficient B cells (Fig 2D and S4B Fig). We could not determine the contribution of either molecule in the maintenance of Mcl-1 expression by tonic BCR signaling due to low expression levels in BCAP- or CD19-deficient cells. We conclude that Mcl-1 expression in response to BCR-initiated signals requires PI3K activation via the involvement of Syk, Btk and BCAP; thereafter, the effects of PI3K are mediated by mTOR.

### mTOR-independent induction of p100 (Nfkb2) expression by the BCR

**Fig 3:**
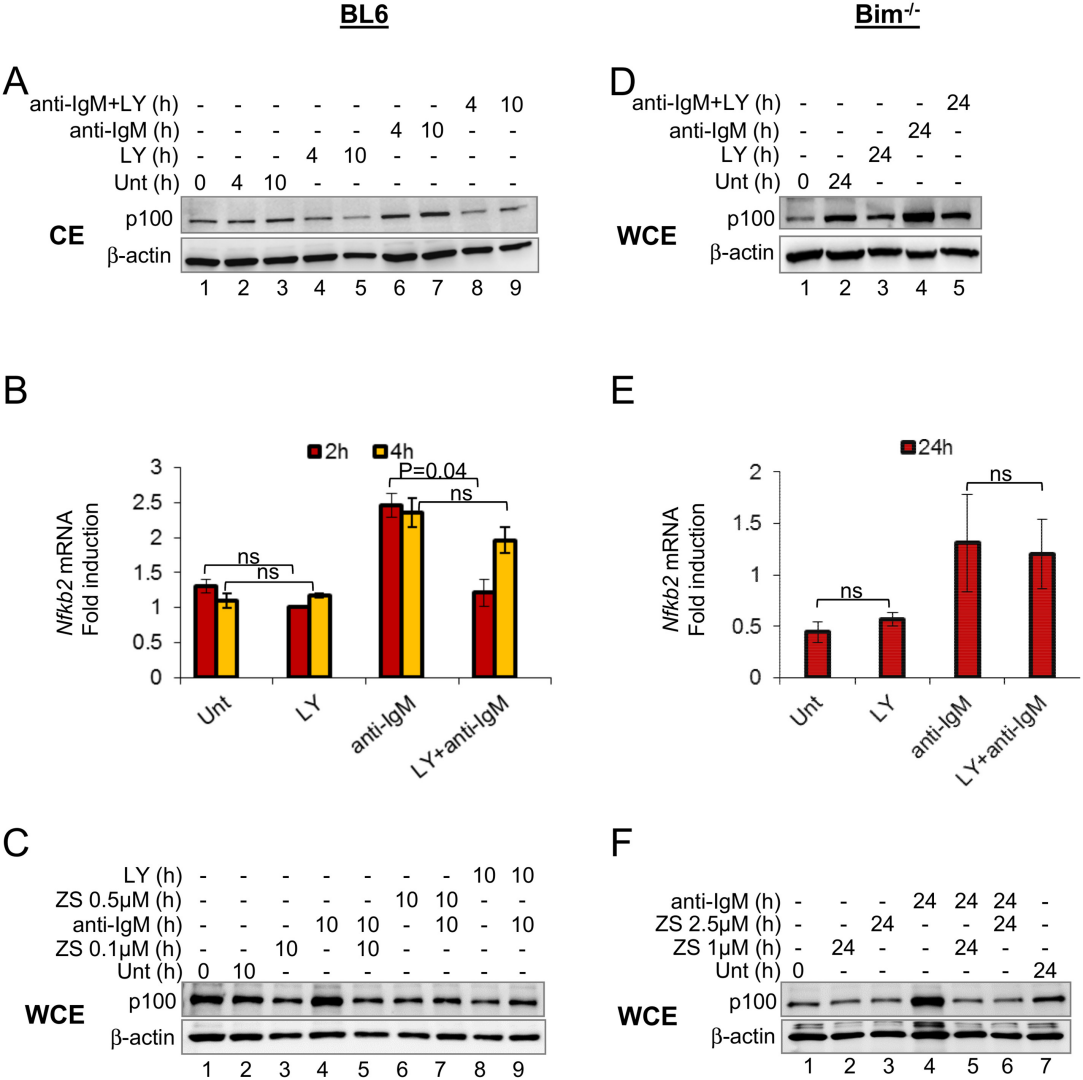
PI3K-dependent *Nfkb2* transcription and p100 protein expression. CD43^-^ B cells from BL6 or Bim^-/-^ mice were incubated for various times with or without anti-IgM F(ab’)_2_ fragment (15ug/ml) in the presence or absence of the PI3K inhibitors LY294002 (20μM) or ZSTK474 (ZS 0.1μM, 0.5μM, 1μM and 2.5μM). (A, C, D, F). Cytosolic (CE) or whole cell (WCE) extracts were fractionated by SDS-PAGE and p100 protein expression was analyzed by immunoblotting. β-actin was used to normalize between samples. (B, E) *Nfkb2* mRNA levels were determined by quantitative RT-PCR and *Nfkb2* mRNA expression was normalized to β-actin. mRNA fold-change (Y axis) was calculated relative to the levels in untreated cells at 0h. The average of 3 independent experiments is shown. Error bars represent the standard error of the mean between experiments with statistical comparison between untreated cells and LY treated or anti-IgM treated with anti-IgM+LY treated cells. P values were calculated using paired TTEST in Microsoft Office Excel (2013) with two tailed distribution. Ns = not significant. Cell viability profiles are noted in S3 Fig.

We found that p100 up-regulation in response to tonic or anti-Ig treatment also required PI3K (Fig 3A) and occurred at a post-transcriptional level (Fig 3B). We could not extend the experiment beyond 10h due to extensive death of LY-treated B cells from BL6 mice (S3A Fig). To circumvent this problem, we used Bim-deficient B cells. 24h *ex vivo* culture of these cells showed the expected two-fold increase in p100 protein (Fig 3D, lanes 1, 2), which we previously inferred to be due to tonic BCR signaling [7]. As observed at earlier time points, p100 up-regulation in response to both tonic and acute BCR signaling was reduced in the presence of LY (Fig 3D, lanes 2–5). *Nfkb2* mRNA expression did not correlate with p100 protein expression in the presence or absence of LY (Fig 3E), suggesting that p100 expression was regulated post-transcriptionally. A different PI3K inhibitor, ZSTK474, affected p100 levels similarly to that observed with LY (Fig 3C and 3F). We conclude that signals from the BCR maintain or induce p100 expression, depending on the strength of the stimulus, via a PI3K-dependent pathway.

**Fig 4:**
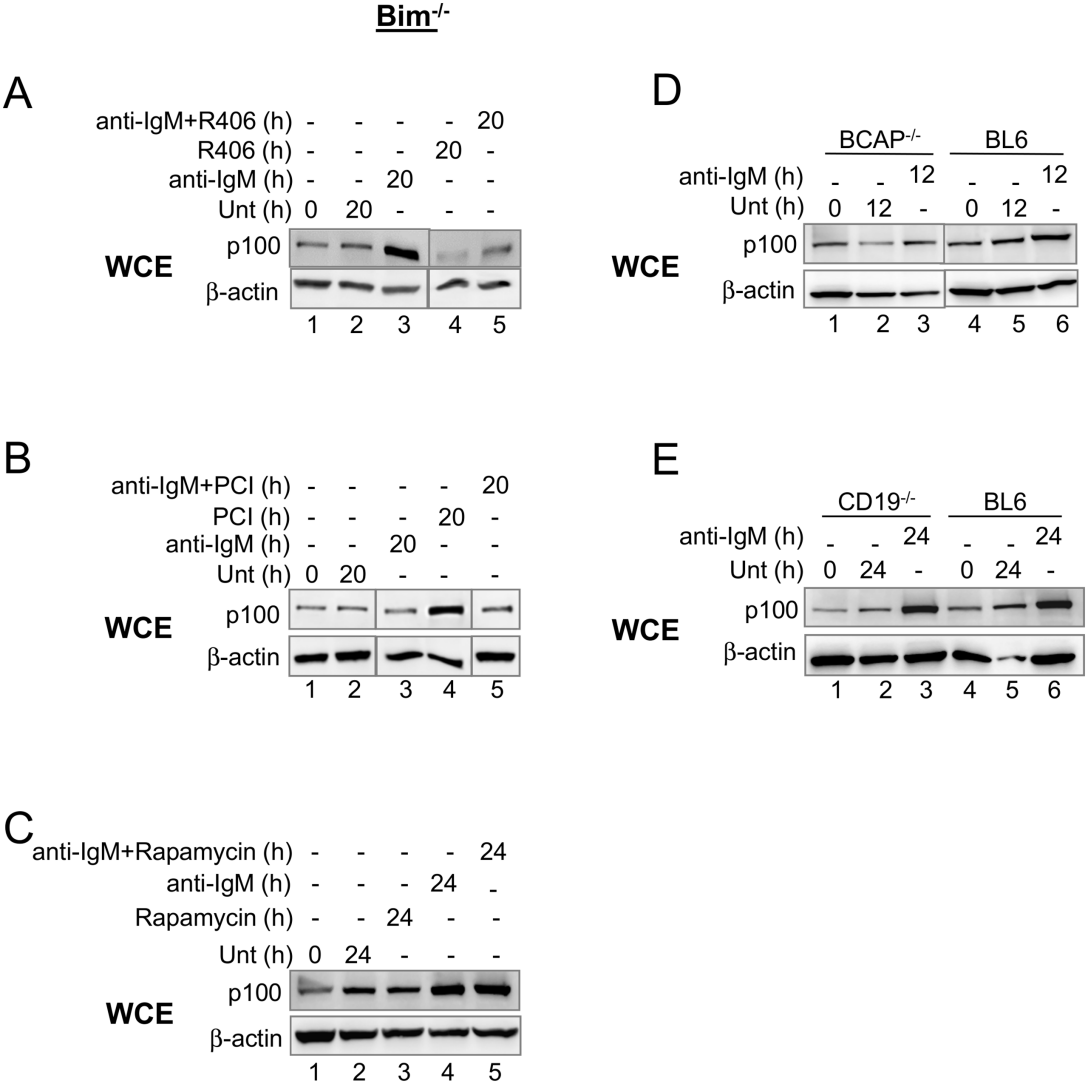
Signaling pathway to BCR-induced p100 expression. (A-C) CD43^-^ splenic B cells from Bim^-/-^ mice were treated with anti-IgM F(ab’)_2_ (15μg/ml) for various times in the presence or absence of Syk kinase inhibitor R406 (4μM), Bruton’s tyrosine kinase inhibitor Ibrutinib (PCI-32765) (20nM), or TORC1 inhibitor rapamycin (50nM). Whole cell extracts were fractionated by SDS-PAGE and p100 proteins were analyzed by immunoblotting. β-actin was used as a loading control to normalize between samples. (D, E) CD43^-^ splenic B cells from BL6, BCAP^-/-^ (D) or CD19^-/-^ (E) mice were cultured for the indicated times with or without anti-IgM F(ab’)_2_ (15μg/ml). Whole cell extracts were prepared and assayed for p100 protein levels by Western blot analysis. β-actin was used as a loading control to normalize between samples. Representative gels from 3 independent experiments are shown. Cell viability is provided in S4 Fig.

We next compared signaling pathways for PI3K-dependent, post-transcriptional induction of Mcl-1 and p100 downstream of the BCR. We found that receptor proximal signaling requirements were similar for p100 and Mcl-1 expression. Specifically, Syk and Btk inhibitors, as well as BCAP-deficiency, but not CD19 deficiency, substantially reduced tonic or anti-IgM-induced p100 expression (Fig 4A, 4B, 4D and 4E). Interestingly, receptor-distal requirement for PI3K differed significantly in one important way. p100 expression in response to BCR was insensitive to rapamycin (Fig 4C) whereas Mcl-1 expression was sensitive to rapamycin (Fig 2B). Taken together, these observations demonstrate that 1) BCR-initiated signals up-regulate both key survival proteins, Mcl-1 and p100, by PI3K-dependent mechanisms, 2) the requirement for BCAP is greater than that for CD19 in transmitting BCR signals to PI3K and 3) distinct post-transcriptional mechanisms regulate Mcl-1 and p100 protein expression. We propose that the close relationship between induction of these proteins by acute BCR stimulation and their maintenance during *ex vivo* culture provides strong evidence in support of tonic BCR signaling.

### mTOR-dependent and independent induction of Mcl-1 by BAFF

**Fig 5:**
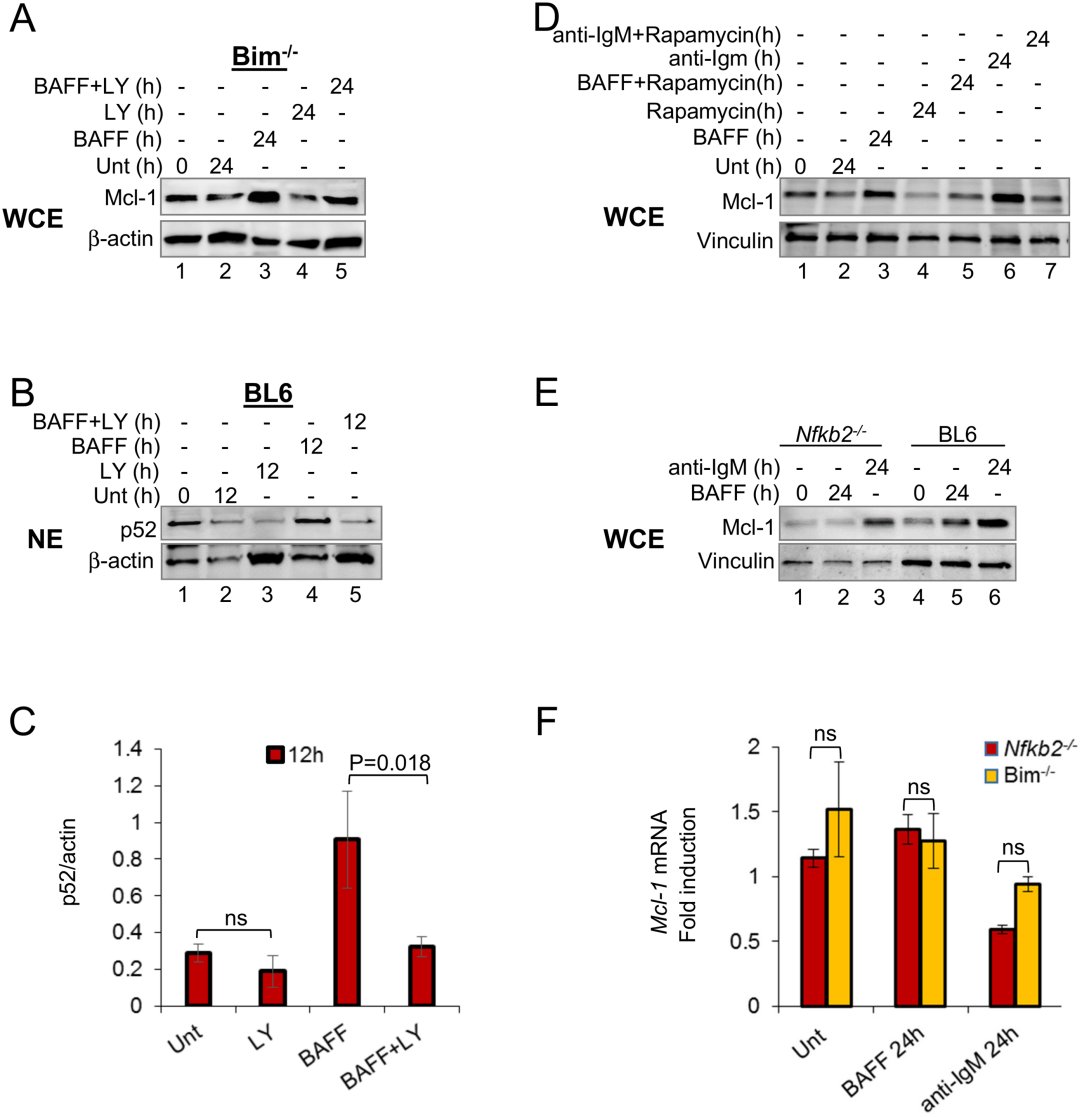
Rapamycin-sensitive and insensitive pathways to Mcl-1 up-regulation by BAFF. (A-D) CD43^-^ splenic B cells from BL6 or Bim^-/-^ mice were treated with BAFF (200ng/ml) for various times in the presence or absence of the PI3K inhibitor LY294002 (20μM) or rapamycin (50nM) as indicated. anti-IgM F(ab’)_2_ (15ug/ml) was included in some experiments as indicated. Whole cell extract (WCE) (A, D) and nuclear extracts (NE) (B) were fractionated by SDS-PAGE and assayed for Mcl-1 or p52 proteins by immunoblotting. β-actin was used to normalize between samples. Gel in part B shows representative Western blot from four independent experiments. p52 levels were quantified as described in the methods section, and the average of four independent experiments is shown in part C. The level of p52 at 0h was assigned the value of 1 (Y axis). (E, F) CD43^-^ splenic B cells from BL6 and Nfkb2^-/-^ mice were left untreated or treated with BAFF (200ng/ml) or anti-IgM F(ab’)_2_ (15μg/ml) for the indicated times. Whole cell extract (WCE) (E) was fractionated by SDS-PAGE and assayed for Mcl-1 protein by immunoblotting and vinculin was used to normalize between samples. (F) Mcl-1 mRNA levels were determined by quantitative RT-PCR normalized to β-actin. The average of 2 independent experiments is shown. P values were calculated using paired TTEST in Microsoft Office Excel (2013) with two tailed distribution. Ns = not significant.

Based on evidence presented above that B cells continuously receive tonic BCR signals, we re-evaluated the effects of *ex vivo* BAFF treatment on Mcl-1 expression and p100 processing. At the crux of this re-evaluation is the idea that B cells treated with BAFF would receive two signals, 1) the constitutive (tonic) signals from the BCR and 2) the active signal from BAFF-bound BAFF-R. Since both BAFF-R and the BCR activate PI3K, we first tested the effects of inhibiting this enzyme during culture of B cells with BAFF. We found that expression of Mcl-1 in response to BAFF was inhibited by blocking PI3K activity with LY (Fig 5A). Likewise, persistent up-regulation of p52 via processing of p100 was abolished in LY-treated cells (Fig 5B and 5C), likely due to lack of p100 synthesis in the absence of tonic BCR-induced PI3K activity. This idea was substantiated by directly quantitating p100 levels in LY-treated cells (S5A and S5B Fig). Reduced levels of Mcl-1 and p52 in BAFF-treated cells cultured with LY correlated with reduced viability of these cells (S5C Fig).

Mcl-1 expression was reduced in cells treated with rapamycin alone (Fig 5D, compare lanes 2, 4) presumably reflecting reduced tonic BCR signaling. However, Mcl-1 levels were higher in BAFF plus rapamycin-treated cells compared to cells treated with rapamycin alone (Fig 5D, lanes 4, 5), suggesting presence of a BAFF-dependent, but rapamycin-independent, pathway for Mcl-1 up-regulation. Previous studies showed that rapamycin did not affect BAFF-dependent B cell survival *ex vivo* [30]. Since Mcl-1 is essential for B cell survival in response to BAFF, we inferred that residual levels of Mcl-1 in BAFF plus rapamycin-treated cells must be sufficient to provide BAFF-dependent survival. Considering that *Nfkb2*^*-/-*^ B cells do not survive in response to BAFF, and that p100 expression was rapamycin-insensitive, we tested Mcl-1 levels in *Nfkb2*^*-/-*^ B cells. We found that BAFF treatment did not induce Mcl-1 expression in these cells (Fig 5E, compare lanes 1, 2 to 4, 5), whereas Mcl-1 induction by the BCR was not affected (Fig 5E, compare lanes 2, 3 to 5, 6). Mcl-1 mRNA levels were comparable between WT and *Nfkb2*^*-/-*^ B cells, thereby ruling out a transcriptional effect to account for these observations (Fig 5F). We propose that the proportion of Mcl-1 induced by BAFF that is rapamycin-insensitive requires Nfkb2. A likely candidate to mediate this effect is Pim-2, a translation-enhancing protein kinase [41] that is transcriptionally induced by p52/RelB [19].

## Discussion

Our studies provide plausible connections between three molecules that are essential for mature B cell survival, the BCR, BAFF-R and Mcl-1. We show that signals initiated at the BCR induce Mcl-1 expression by a PI3K-dependent, rapamycin-sensitive pathway. The fact that maintenance of Mcl-1 expression in the absence of ectopic activation requires many of the same cytosolic components as those that are required in acutely stimulated cells is consistent with the idea that B cells continuously receive low-grade BCR signals. We propose that this reflects tonic BCR signaling. Acute or tonic BCR signals also induce p100 expression via a PI3K-dependent pathway. Unlike Mcl-1, however, p100 induction is rapamycin-insensitive. Moreover, since p100 mRNA levels are unaffected by PI3K inhibition, we surmise that PI3K promotes translation of p100 mRNA to up-regulate protein expression. These observations reveal a role for TORC1-independent translational pathway downstream of the BCR. The importance of p100 production in response to tonic BCR signaling is evident when considering the effects of BAFF/BAFF-R on B cell survival as described below.

Woodland *et al*, previously showed that B cells lacking Mcl-1 do not respond to BAFF treatment *ex vivo*. Consistent with their observations, we found that BAFF treatment induced Mcl-1 in B cells. However, we interpreted these results in the context of co-existing tonic BCR signals that also induce Mcl-1 expression. That is, Mcl-1 expression in BAFF-treated B cells is the consequence of both BCR- and BAFF-R-initiated signals. Knowing that BCR-dependent Mcl-1 expression was rapamycin-sensitive, we attempted to distinguish between these pathways by including rapamycin during BAFF treatment. Despite abrogating Mcl-1 expression due to tonic BCR signaling, we detected residual Mcl-1 induction under these conditions, suggesting that BAFF treatment induced Mcl-1 by a rapamycin-insensitive pathway independent of BCR signaling to Mcl-1. Though the effect of either the BCR pathway or the BAFF-R pathway is small, we suggest that cumulative Mcl-1 levels exceed a threshold required for B cell survival. We next sought to identify the rapamycin-independent pathway by which BAFF induced Mcl-1. Because B cells deficient in either Mcl-1 or Nfkb2 do not respond to BAFF, we hypothesized that these two gene products may be causally linked to BAFF-dependent B cell survival. This idea was corroborated by the observation that *Nfkb2*^*-/-*^ B cells did not induce Mcl-1 expression in response to BAFF. One possibility was that Nfkb2-derived non-classical NF-κB regulated Mcl-1 transcription. However, Mcl-1 mRNA levels were comparable in WT and *Nfkb2*^*-/-*^ B cells. Therefore, we propose that non-classical NF-κB-induced genes regulate Mcl-1 expression in BAFF-treated cells. This model accounts for rapamycin-insensitive Mcl-1 expression by BAFF since neither BCR-induced p100 expression nor BAFF-dependent p100 processing require mTOR. A possible non-classical NF-κB target gene that could mediate this effect is Pim-2, as it is known to regulate translation [41,42] and, in special circumstances, has been implicated in the expression of Mcl-1 itself [43,44].

In summary, our working model for the dual receptor requirement for B cell survival is as follows. BCR- initiated signals induce Mcl-1 by a rapamycin-sensitive pathway and p100 by a rapamycin-insensitive pathway. BAFF treatment adds to the sub-threshold levels of Mcl-1 induced by tonic BCR signals to those required for B cell survival. It does so by converting p100 produced by tonic BCR signals to an active transcription factor (presumably p52/RelB) that induces expression of translation-regulating genes such as Pim-2. The notion of two receptors that generate sub-threshold levels of a survival factor is attractive for several reasons. First, it affords survival flexibility such that, depending on the physiological circumstance, each receptor may play a bigger or a smaller role. Second, the model accounts for several earlier observations that indicate that BAFF levels are limiting *in vivo*. Third, proposed roles for rapamycin-sensitive and insensitive pathways provide an explanation for the observation that BAFF responsiveness is abrogated only in rapamycin-treated Pim-2-deficient B cells.

We note that this model appears deficient in explaining why BAFF-dependent augmentation of B cell survival *ex vivo* is unaffected by rapamycin treatment, which abrogates tonic BCR signals to Mcl-1. One possibility is that Mcl-1 expression via an Nfkb2-dependent mechanism dominates the response at the concentrations of BAFF typically used for *ex vivo* studies, thus diminishing the role of tonic BCR-induced Mcl-1. Additionally, BAFF could activate other survival mechanisms beyond Mcl-1 induction. This does not rule out a role for the BCR *ex vivo*, which likely contributes in other ways beyond Mcl-1 induction to maintain B cell viability, as is evident from the attenuated survival response to BAFF in the presence of a Syk inhibitor.

An obvious role for the tonic BCR signaling in this experimental paradigm is to induce p100 via a rapamycin-independent mechanism in order to maximally utilize BAFF-R-initiated survival signaling. The lack of response of Nfkb2^-/-^ B cells to BAFF demonstrates this essential role of p100 in mediating cell survival *ex vivo*. In part this is mediated by inducing Mcl-1 expression. Thus, rapamycin treatment of Pim2-deficient B cells *ex vivo* blocks both BCR- and p100-dependent Mcl-1 expression, resulting in accentuated cell death. Additionally, tonic BCR signals may induce pro-survival NF-κB activity to maintain B cell viability *in vivo* and in our cell culture studies. It is not our intention to imply that the Mcl-1/p100 interplay proposed here is the complete story of how the BCR and BAFF-R program B cell survival. Rather, we used these two essential molecules to probe mechanisms that maintain peripheral B cells and by doing so uncovered novel rapamycin-dependent and -independent pathways emanating from the BCR that work with the BAFF-R for B cell survival.

## Materials and Methods

### Mice

BL6, Bim^-/-^, BCAP^-/-^ and *Nfkb2*^-/-^ mice were maintained in the animal facility of the National Institute on Aging. CD19^-/-^ [35] were described previously. Eight- to 10-week-old mice were used for all experiments. The studies were carried out in accordance with the recommendations in the Guide for the Care and Use of Laboratory Animals (NRC 2010). Mice were euthanized with carbon dioxide and tissues harvested for analysis. The protocol was approved by the Animal Care and Use Committee of the NIA Intramural Research Program, NIH. This program is fully accredited by the Association for Assessment and Accreditation of Laboratory Animal Care International (AAALAC) (File 000401), registered by the United States Department of Agriculture (51-F-0016) and maintains an assurance with the Public Health Service (A4149-01).

### B cell isolation

Primary B lymphocytes were isolated from spleen using AutoMACS depletion with anti-CD43-conjugated microbeads (Miltenyi Biotec, Auburn, CA) according to the manufacturer’s instructions. Cells were cultured in RPMI 1640 medium (GIBCO, Carlsbad, CA) containing 10% FBS, 50μM β-mercaptoethanol, 1% L-glutamine and 1% penicillin-streptomycin solution. B cell purity was > 95% based on flow cytometric analysis following staining with anti-CD19 (BD Bioscience, San Jose, CA). In some experiments, B cells were cultured with mBAFF (R&D Systems, Minneapolis, MN), or treated with goat anti-mouse IgM F(ab’)_2_ (Jackson ImmunoResearch, West Grove, PA). PI3K inhibitor, LY294002 (Calbiochem, La Jolla, CA), was used at final concentration of 20μM, ZSTK474 (Selleckchem, Houston, TX) was used at different concentrations and Btk was inhibited by the addition of 20nM PCI-32765 (Selleckchem, Houston, TX). Syk inhibitor, R406 (Selleckchem, Houston, TX), was used at a final concentration of 4μM and 50μM of the mTOR inhibitor Rapamycin was used.

### Nuclear and cytosolic protein fractionation

Nuclear and cytoblasmic extracts were performed as previously described [7].

### SDS-PAGE and immunoblot analysis

Cells were collected and washed with cold PBS (GIBCO, Carlsbad, CA), then lysed in RIPA lysis buffer (Santa Cruz Biotechnology, Santa Cruz, CA). Proteins were quantified and separated as previously described [7].

### Real-time PCR

Total RNA was extracted from splenic B cells using the RNeasy Mini Kit (Qiagen, Valencia, CA) according to the maufacturer’s protocol. Total RNA was used for first-strand cDNA synthesis using random hexamer primers and Superscript RT III (Invitrogen, Carlsbad, CA). Quantitative real-time PCR was performed using the ABI Prism7000 (Applied Biosystems, Carlsbad, CA) with SYBR Green Supermix (Bio-Rad, Hercules, CA) according to the manufacturer’s instructions. Abundance of *Nfkb2* or Mcl-1 sequence in the sample relative to the β-actin sequence was determined as described previously [45] where the relative abundance of the target sequence is 2^(Ct[β-actin]- Ct [Nfkb2])^ and Ct is the cycle number at which the samples reach a threshold value in a range where PCR amplification is exponential. Primer sequences used include: Nfkb2, 5’-GCCGGAAGACCTATCCTACTGTCA; 3’-TTCGTCACAAGTCTCAACCCTCACA; Mcl-1 5’- GGTGCCTTTGTGGCCAAACACTTA; 3’- TTGTTTCTCCGACCCTACCCAAAC; β-actin, 5’- CCACGATGGAGGGGA ATACAG; 3’-GTTGCTTCCTCGACGTTTCTTCGA.

### Flow cytometry

Splenic B cells obtained from wild type or mutant mice were made into single-cell suspensions in PBS supplemented with 0.1% BSA. Cells were then stained with FITC-conjugated anti-mouse CD19 antibody (BD Biosciences, San Jose, CA) for 20 min at 4°C. Data were collected on a FACSCalibur (BD Biosciences, San Jose, CA) and analyzed using FlowJo software. Cell viability was determined by propidium iodide staining and flow cytometry.

## Supporting Information

S1 Fig (A) Total cell numbers and CD43^-^ splenic B cells from *Nfkb2*^-/-^ and BL6 mice. (B) CD43^-^ splenic B cells from *Nfkb2*^-/-^ and BL6 mice were cultured *ex vivo* at 37°C with or without BAFF (200ng/ml) for the indicated times. Viability was determined by propidium iodide staining and flow cytometry. Error bars represent the standard error of the mean between experiments. Data are representative of four independent experiments.
(PDF)

S2 Fig (A) CD43^-^ splenic B cells from Bim^-/-^ mice were cultured *ex vivo* in the presence or absence of cycloheximide (10μg/ml); anti-IgM F(ab’)_2_ (15ug/ml) was included in some experiments as indicated. Whole cell extracts were fractionated by SDS-PAGE and Mcl-1 protein expression was analyzed by immunoblotting. β-actin was used to normalize between samples and the gel is a representative Western blot of two independent experiments. (B and C) CD43^-^ splenic B cells from Bim^-/-^ mice were pre-cultured *ex vivo* overnight to remove any pre-bound BAFF, then cells were incubated with LY294002 (20μM) in the presence or absence of BAFF (200ng/ml) for the indicated times. (B) Whole cell extracts were fractionated by SDS-PAGE and p100, p52 and Mcl-1 protein expression were analyzed by immunoblotting. β-actin was used to normalize between samples and the gels show a representative Western blot from two independent experiments. (C) Cell viability was determined by propidium iodide staining and flow cytometry. Error bars represent standard error of the mean between experiments. Student TTEST was performed in Microsoft Office Excel (2013) to compare treated to untreated cells at 24h and P values were not significant. Data are representative of two independent experiments.
(PDF)

S3 Fig (A-D) CD43^-^ splenic B cells from BL6 and Bim^-/-^ mice were cultured *ex vivo* in the presence or absence of the PI3K inhibitor LY294002 (20μM) or ZSTK474 (ZS, 1μM and 2.5μM); anti-IgM F(ab’)_2_ (15ug/ml)) was included in some experiments as indicated. Cell viability was determined by propidium iodide staining and flow cytometry. Error bars represent standard error of the mean between experiments with statistical comparison between untreated cells and LY treated or anti-IgM treated with anti-IgM+LY treated cells. P values were calculated using paired TTEST in Microsoft Office Excel (2013) with two tailed distribution. P values are noted only when statistical comparison was significant. Data are representative of two independent experiments.
(PDF)

S4 Fig Cell viability with or without anti-IgM. CD43^-^ splenic B cells from BL6, BCAP^-/-^ (A) or CD19^-/-^ (B) mice were stimulated with anti-IgM F(ab’)_2_ (15μg/ml) for the indicated times. Cell viability was determined by propidium iodide staining and flow cytometry. Data are representative of three (A) or two (B) independent experiments. Error bars represent standard error of the mean between experiments. Student TTEST was performed in Microsoft Office Excel (2013) to compare the viability of B cells from BCAP^-/-^ (A) or CD19^-/-^ (B) to BL6 at 12h or 24h respectively; no significant differences were observed.
(PDF)

S5 Fig CD43- splenic B cells from BL6 mice were cultured ex vivo in the presence or absence of the PI3K inhibitor LY294002 (20μM); BAFF (200ng/ml) was included in some experiments as indicated. Cytoplasmic extracts were fractionated by SDS-PAGE and p100 protein expression was analyzed by immunoblotting. β-actin was used to normalize between samples. (B) p100 levels were quantified, normalized to β-actin and the average values are shown in the right panel; the p100 level at 0h in BL6 cells was assigned the value of 1 (Y axis). (C) Viability of B cells was determined by flow cytometry. Error bars represent standard error of the mean between experiments. Data are the average of three independent experiments. P values were calculated using paired TTEST in Microsoft Office Excel (2013) with two tailed distribution.
(PDF)
