# Supplementary figures and images for: mTOR-Dependent and Independent Survival Signaling by PI3K in B Lymphocytes
Source: PLoS One. 2016 Jan 19;11(1):e0146955. doi: 10.1371/journal.pone.0146955 (PMC4718598; doi:10.1371/journal.pone.0146955)

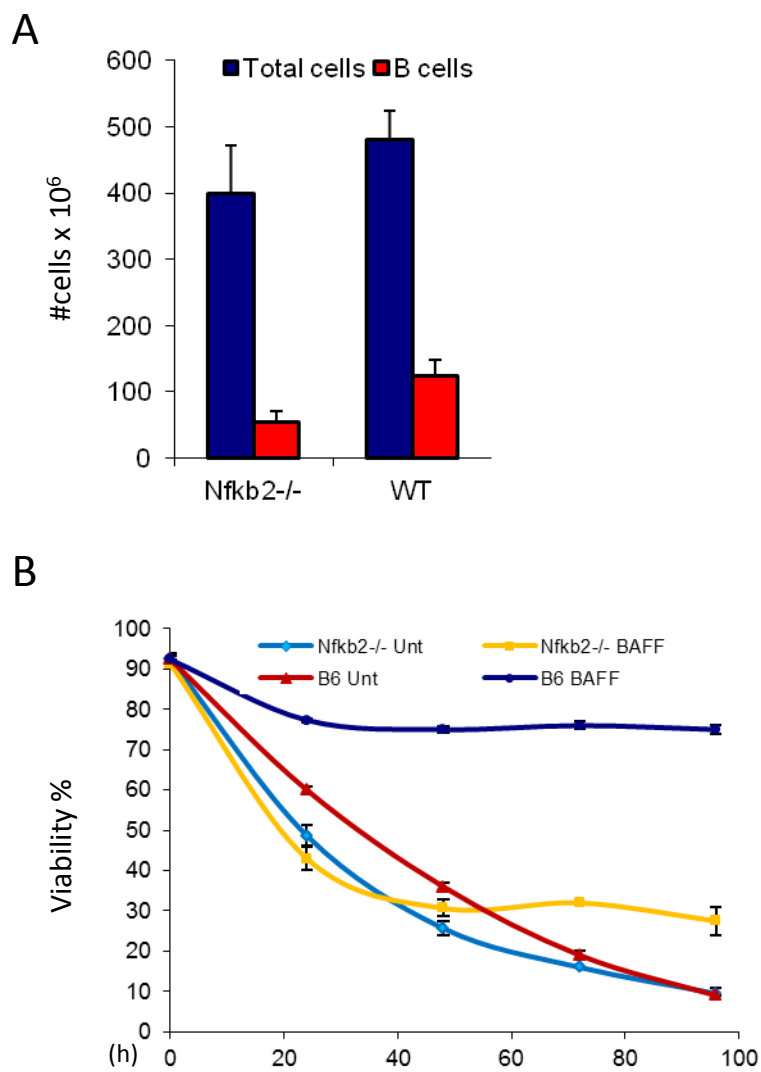

Supplement: S1 Fig — (A) Total cell numbers and CD43- splenic B cells from Nfkb2-/- and BL6 mice. (B) CD43- splenic B cells from Nfkb2-/- and BL6 mice were cultured ex vivo at 37°C with or without BAFF (200ng/ml) for the indicated times. Viability was determined by propidium iodide staining and flow cytometry. Error bars represent the standard error of the mean between experiments. Data are representative of four independent experiments. (PDF) [file pone.0146955.s001.pdf]

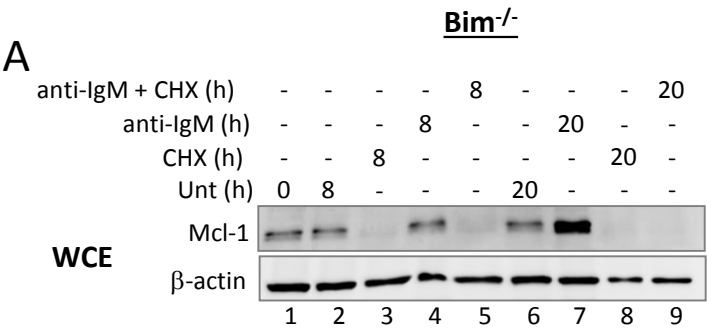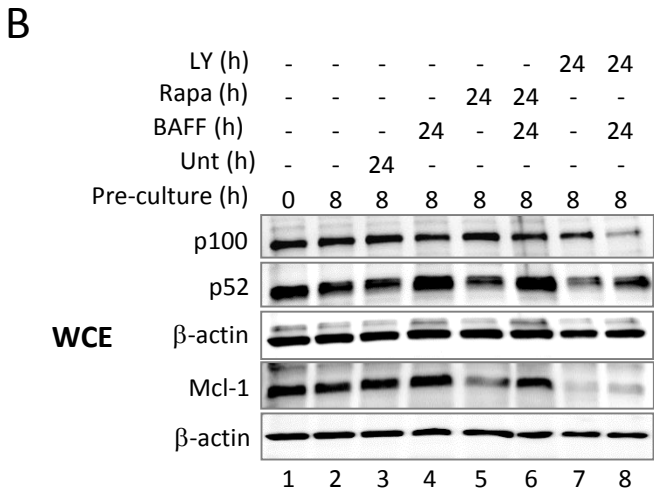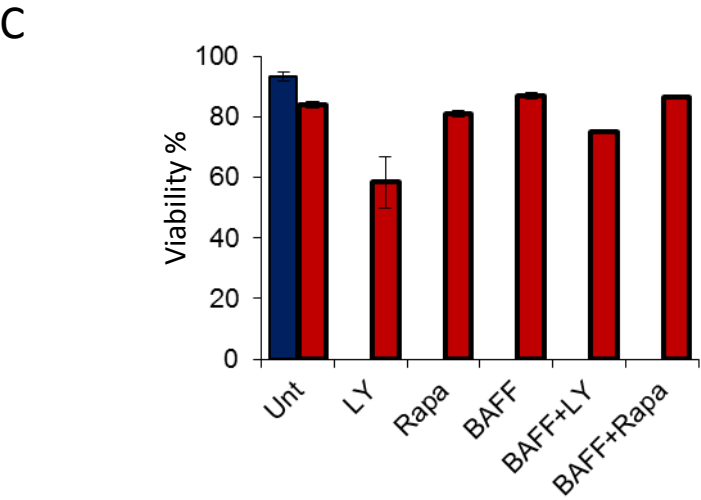

Supplement: S2 Fig — (A) CD43- splenic B cells from Bim-/- mice were cultured ex vivo in the presence or absence of cycloheximide (10μg/ml); anti-IgM F(ab’)2 (15ug/ml) was included in some experiments as indicated. Whole cell extracts were fractionated by SDS-PAGE and Mcl-1 protein expression was analyzed by immunoblotting. β-actin was used to normalize between samples and the gel is a representative Western blot of two independent experiments. (B and C) CD43- splenic B cells from Bim-/- mice were pre-cultured ex vivo overnight to remove any pre-bound BAFF, then cells were incubated with LY294002 (20μM) in the presence or absence of BAFF (200ng/ml) for the indicated times. (B) Whole cell extracts were fractionated by SDS-PAGE and p100, p52 and Mcl-1 protein expression were analyzed by immunoblotting. β-actin was used to normalize between samples and the gels show a representative Western blot from two independent experiments. (C) Cell viability was determined by propidium iodide staining and flow cytometry. Error bars represent standard error of the mean between experiments. Student TTEST was performed in Microsoft Office Excel (2013) to compare treated to untreated cells at 24h and P values were not significant. Data are representative of two independent experiments. (PDF) [file pone.0146955.s002.pdf]

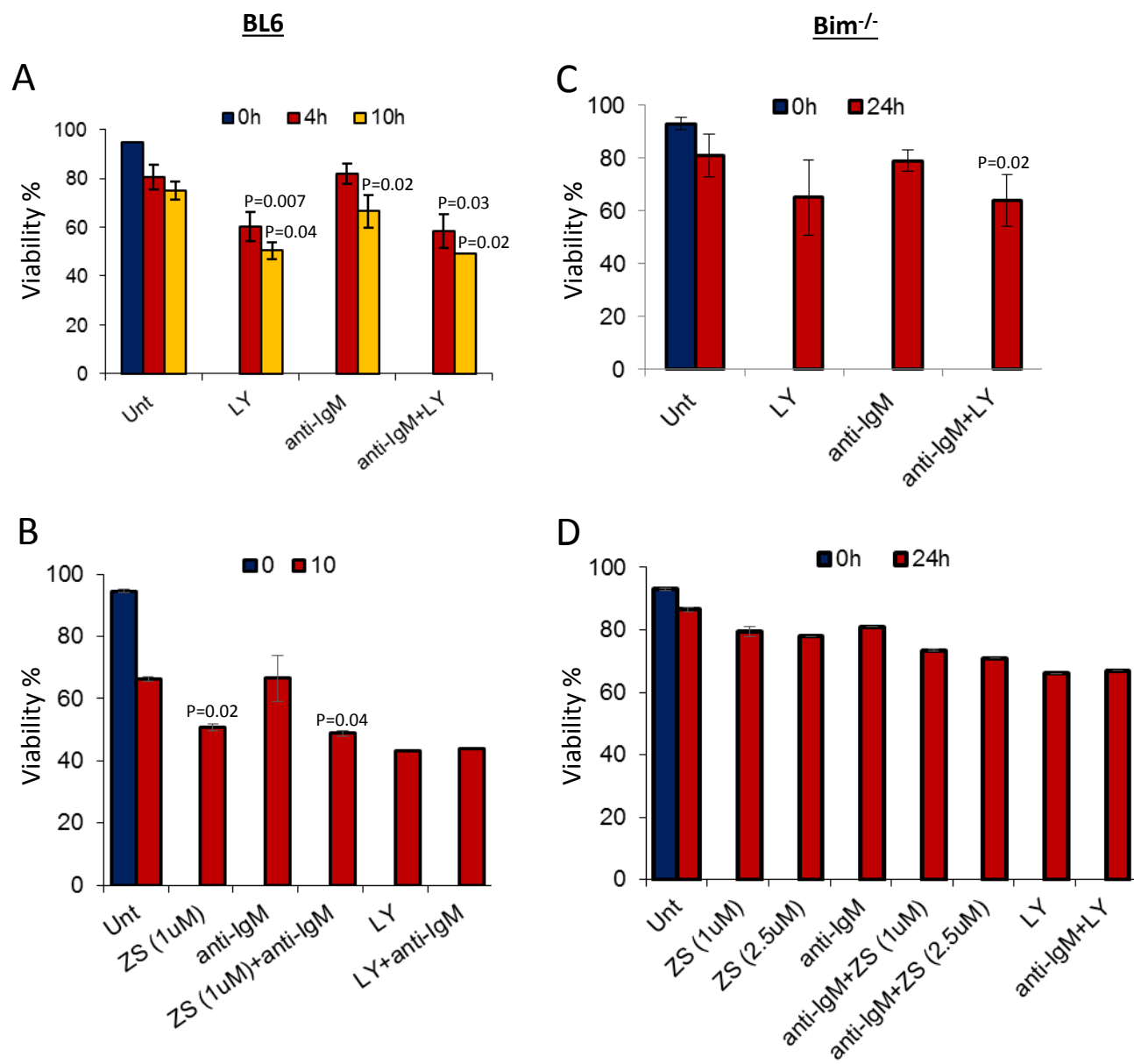

Supplement: S3 Fig — (A-D) CD43- splenic B cells from BL6 and Bim-/- mice were cultured ex vivo in the presence or absence of the PI3K inhibitor LY294002 (20μM) or ZSTK474 (ZS, 1μM and 2.5μM); anti-IgM F(ab’)2 (15ug/ml)) was included in some experiments as indicated. Cell viability was determined by propidium iodide staining and flow cytometry. Error bars represent standard error of the mean between experiments with statistical comparison between untreated cells and LY treated or anti-IgM treated with anti-IgM+LY treated cells. P values were calculated using paired TTEST in Microsoft Office Excel (2013) with two tailed distribution. P values are noted only when statistical comparison was significant. Data are representative of two independent experiments. (PDF) [file pone.0146955.s003.pdf]

A

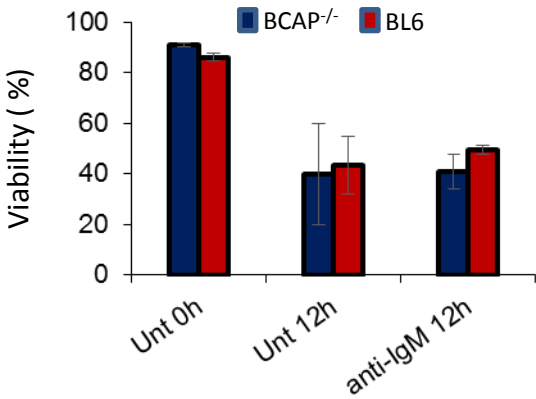

B

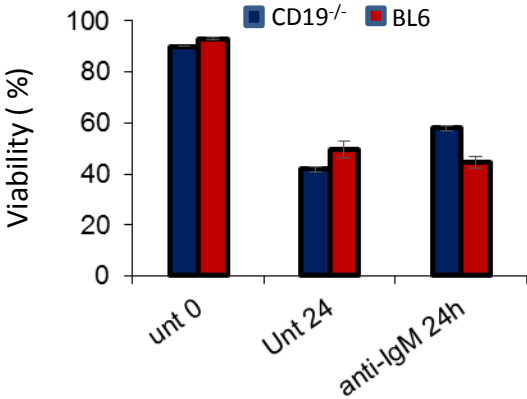

Supplement: S4 Fig — CD43- splenic B cells from BL6, BCAP-/- (A) or CD19-/- (B) mice were stimulated with anti-IgM F(ab’)2 (15μg/ml) for the indicated times. Cell viability was determined by propidium iodide staining and flow cytometry. Data are representative of three (A) or two (B) independent experiments. Error bars represent standard error of the mean between experiments. Student TTEST was performed in Microsoft Office Excel (2013) to compare the viability of B cells from BCAP-/- (A) or CD19-/- (B) to BL6 at 12h or 24h respectively; no significant differences were observed. (PDF) [file pone.0146955.s004.pdf]

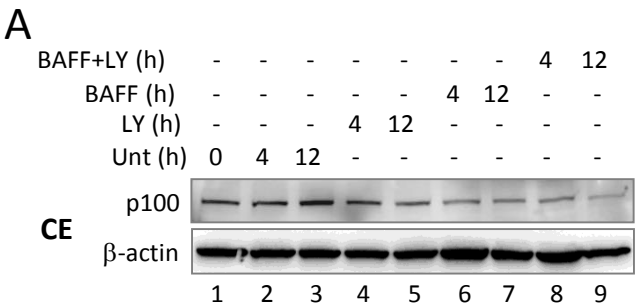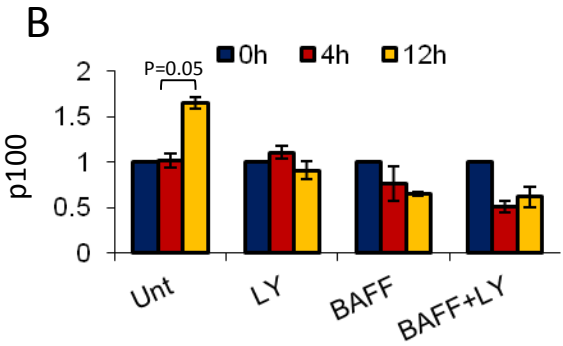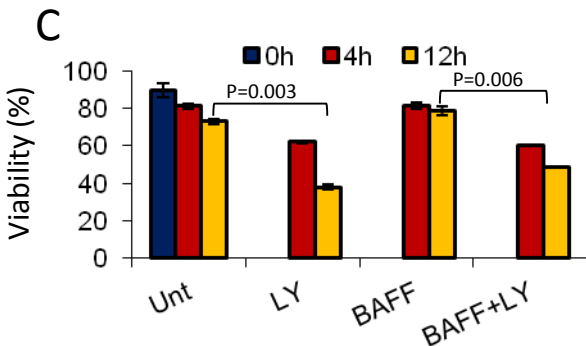

Supplement: S5 Fig — Cytoplasmic extracts were fractionated by SDS-PAGE and p100 protein expression was analyzed by immunoblotting. β-actin was used to normalize between samples. (B) p100 levels were quantified, normalized to β-actin and the average values are shown in the right panel; the p100 level at 0h in BL6 cells was assigned the value of 1 (Y axis). (C) Viability of B cells was determined by flow cytometry. Error bars represent standard error of the mean between experiments. Data are the average of three independent experiments. P values were calculated using paired TTEST in Microsoft Office Excel (2013) with two tailed distribution. (PDF) [file pone.0146955.s005.pdf]
